# The complete chloroplast genome of *Changium smyrnioides* Wolff

**DOI:** 10.1080/23802359.2019.1688715

**Published:** 2019-11-18

**Authors:** Zhenzhen Bao, Ziyan Zhu, Yanan Gai

**Affiliations:** a School of Pharmacy, Jiangsu Health Vocational College, Nanjing, Jiangsu, P. R. China;; b School of Traditional Chinese Pharmacy, China Pharmaceutical University, Nanjing, Jiangsu, P. R. China;; c Institute of Botany, Jiangsu Province and Chinese Academy of Sciences, Nanjing, Jiangsu, P. R. China

**Keywords:** *Changium smyrnioides*, complete chloroplast genome, phylogenetic analysis

## Abstract

*Changium smyrnioides* Wolff, which could only be found in Eastern China, is a monotypic species of the genus Changium Wolff. In this study, the complete chloroplast genome sequence of *C. smyrnioides* was assembled and characterized by the 42.33 M high-throughput sequencing data. The chloroplast genome was 155,221 bp in length, consisting of large single-copy (LSC) and small single-copy (SSC) regions of 84,793 bp and 17,828 bp, respectively, which were separated by a pair of 26,300 bp inverted repeat (IR) regions. The genome is predicted to contain 131 genes, including 8 rRNA genes, 37 tRNA genes, and 86 protein-coding genes. The overall GC content of the genome is 37.7%. A phylogenetic tree reconstructed by 37 chloroplast genomes reveals that *Chuanminshen violaceum* is mostly related species to *C. smyrnioides*. The work reported here is the first complete chloroplast genome of *C. smyrnioides* which may provide useful information to the evolution of *Changium* genus.

*Changium smyrnioides*, listed in the endangered species of China, is a monotypic species of the genus *Changium*. It could only be found in eastern China, especially at an altitude of 50–400 m above sea level (Wang et al. 2017). As a great valued medicinal plant, the dry root of *C. smyrnioides* is widely used for treating cough, vomiting, nausea, xerostomia, megrim, furunculosis (Lei et al. 2019). Up to now, more than 100 chemical constituents have been isolated and identified from *C. smyrnioides*, including simple phenylpropanoids, coumarins, volatile oils, fatty acids, polysaccharides, micro-elements, and other compounds (Lei et al. 2019). However, there have been still no reports about the chloroplast (cp) genome information of *C. smyrnioides* yet. In this study, the complete cp genome of *C. smyrnioides* was determined using high-throughput sequencing technology, which will provide informatics data for the phylogeny of Changium genus and further research on Umbelliferae.

The fresh leaves of *C. smyrnioides* were collected from Jurong, Jiangsu, China (31°94′N, 119°17′E) and then the species were stored in Institute of Botany, Jiangsu Province and Chinese Academy of Sciences with the accession number of MDS20190815BZZ-6. FastPure Plant DNA Isolation Mini Kit was used for total genomic DNA extraction (Vazyme, Nanjing, China). The whole-genome sequencing was then conducted by Hefei Biodata Biotechnologies Inc. (Hefei, China) with Illumina Hiseq platform. SPAdes assembler 3.10.0 was used for assembly the filtered sequences (Anton et al. 2012). Annotation was performed using the DOGMA and BLAST searches (Wyman et al. 2004). At last, the cp genome of *C. smyrnioides* was determined to comprise a 155,221 bp double-stranded, circular DNA (GenBank accession no. MN092718), which containing two inverted repeat (IR) regions of 26,300 bp, separated by large single-copy (LSC) and small single-copy (SSC) regions of 84,793 bp and 17,828 bp, respectively. The overall GC content of *C. smyrnioides* cp genome is 37.7% and the corresponding values in LSC, SSC, and IR regions are 35.9, 31.4, and 42.9%, respectively. The cp genome was predicted to contain 131 genes, including 86 protein-coding genes, 37 tRNA genes, and 8 rRNA genes. Five protein-coding genes, seven tRNA genes, and four rRNA genes were duplicated in IR regions. This somewhat in accordance with the cp genome of *Sorbus folgneri* (Qiu et al. 2019). In addition, 19 genes contained two exons and four genes (clpP, ycf3, and two rps12) contained thee exons which in accordance most of the plant cp genomes (Han et al. 2019; Li et al. 2019; Yi et al. 2019).

To investigate its taxonomic status, alignment was performed on the 12 cp genome sequences using MAFFT v7.307, and a maximum likelihood (ML) tree was constructed by FastTree version 2.1.10 (Price et al. 2010; Kazutaka and Standley 2013). As a result, *Chuanminshen violaceum* is the most related species to *C. smyrnioides*, with bootstrap support values of 100% (Figure 1). The complete cp genome sequence of *C. smyrnioides* will provide a useful resource for the conservation genetics of this species as well as for the phylogenetic studies of *Changium* genus.

**Figure 1.**
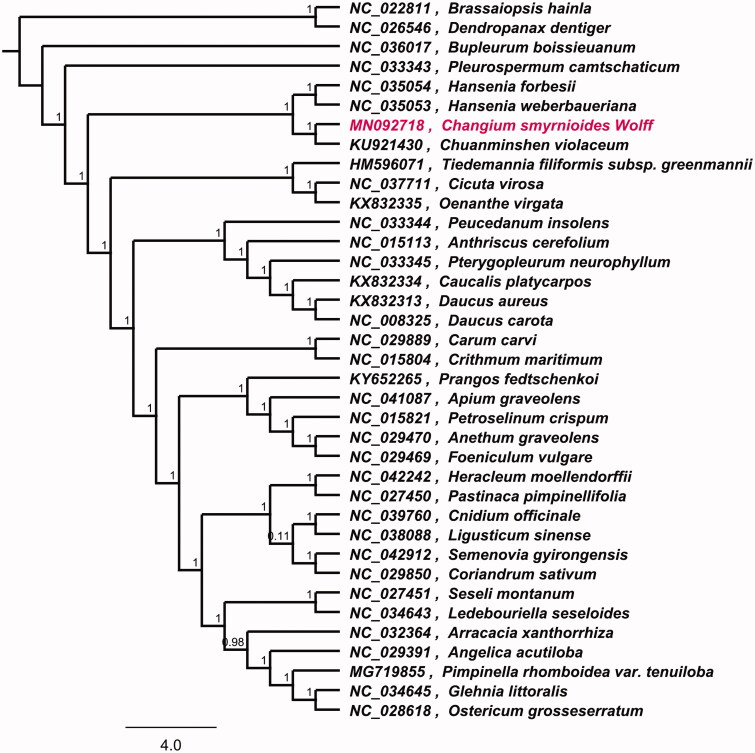
Phylogenetic tree inferred by maximum likelihood (ML) method based on 37 representative species. A total of 1000 bootstrap replicates were computed and the bootstrap support values are shown at the branches. GeneBank accession numbers were shown in Figure 1.
